# Safety Profile of Chimeric Antigen Receptor T-cell Therapies in Diffuse Large B-cell Lymphoma: An Analysis of Real-World Adverse Event Reporting From the FDA Adverse Event Reporting System

**DOI:** 10.1016/j.mayocpiqo.2026.100758

**Published:** 2026-09-23

**Authors:** Magdi Elsallab, Matthew Lunning, Moataz Ellithi, Farah Toron, Robert Braun, Lin Wang, Pearl Wang, Maxwell Jones, Marcela V. Maus

**Affiliations:** a Cellular Immunotherapy Program, Massachusetts General Hospital Cancer Center, Boston, MA; b Harvard Medical School, Boston, MA; c University of Nebraska Medical Center, Nebraska Medical Center, Omaha, NE; d Bristol Myers Squibb, Uxbridge, United Kingdom; e Bristol Myers Squibb, Neuchâtel, Switzerland; f Bristol Myers Squibb, NJ; g EVERSANA, Ontario, Canada

## Abstract

**Objective:**

To compare the real-world safety profiles of lisocabtagene maraleucel (liso-cel) and axicabtagene ciloleucel (axi-cel) in patients with diffuse large B-cell lymphoma, leveraging data from the United States Food and Drug Administration Adverse Event Reporting System (FAERS) database.

**Patients and Methods:**

Case reports submitted to FAERS (Q4 2017 to Q3 2024) were evaluated if liso-cel or axi-cel was identified as the primary suspect therapy for adverse events of interest such as cytokine release syndrome (CRS), immune effector cell–associated neurotoxicity syndrome (ICANS), cytopenia, and infections. Disproportionality analyses compared AE frequencies using reporting odds ratios (ROR). Concomitant treatment use rates were captured to assess the need for supportive medications.

**Results:**

Among 3251 diffuse large B-cell lymphoma-related reports, liso-cel was the primary therapy in 232 cases compared with 3019 attributed to axi-cel. Liso-cel had significantly lower RORs (95% CI) for CRS (0.68; 0.52-0.89), ICANS (0.62; 0.47-0.82), and cytopenia (0.41; 0.24-0.71) vs axi-cel; infection rates were similar (0.73; 0.40-1.33). Fewer patients used concomitant treatments with liso-cel than axi-cel (49 [21.1%] vs 1019 [33.8%]; *P*<.001), particularly corticosteroids, azoles, and levetiracetam. Tocilizumab use was similar (8 [3.5%] liso-cel vs 97 [3.2%] axi-cel), and intravenous immunoglobulin use was rare (<1%).

**Conclusion:**

Liso-cel reported a more favorable safety profile than axi-cel, with fewer reports of CRS, ICANS, cytopenia, and the need for supportive medications. Although efficacy outcomes were not analyzed, safety outcomes are consistent with previously published indirect treatment comparisons and can inform evidenced-based decision-making of clinicians.

Chimeric antigen receptor (CAR) T-cell therapies are integral in the treatment of diffuse large B-cell lymphoma (DLBCL) therapies.^1^, ^2^, ^3^, ^4^ CAR T-cell therapies such as lisocabtagene maraleucel (liso-cel) and axicabtagene ciloleucel (axi-cel) have reported substantial efficacy in clinical trials, resulting in durable remissions and significant improvements in patient outcomes.^5^^,^^6^ CAR T-cell therapies are associated with potential adverse events (AEs) that may impact patient safety and management.^7^ The most commonly observed AEs associated with CAR T-cell therapy are cytokine release syndrome (CRS) and immune effector cell–associated neurotoxicity syndrome (ICANS). Other AEs such as cytopenia and infections also contribute to significant patient morbidity and mortality, and warrant monitoring.^7^^,^^8^

Safety and efficacy of CAR T-cell therapies were initially established through prospective clinical trials; however, these trials are often limited by relatively small sample sizes, restrictive eligibility criteria, and short follow-up duration, which may not fully capture the spectrum or timing of AEs. In addition, rare AEs are not captured in clinical trials. As CAR T-cell therapies are increasingly administered across various patient populations and clinical settings,^9^, ^10^, ^11^ a comprehensive understanding of their safety profile is critical to informing clinical decision-making and optimizing patient management.

Postmarketing surveillance databases such as the United States Food and Drug Administration (FDA) Adverse Event Reporting System (FAERS) provide a unique opportunity to monitor and evaluate AEs in a real-world setting by capturing data over a more extended period from a more diverse patient population than those typically available in clinical trials.^12^ This long-term, large-scale surveillance enables the identification of rare, delayed, or previously underreported AEs that may not have emerged during pre-approval clinical trials.^12^

The objective of this study was to investigate the safety profile of CAR T-cell therapies, specifically CRS, ICANS, cytopenia, and infections, in patients with DLBCL by analyzing FAERS data from case reports involving liso-cel and axi-cel. The insights gained from this analysis will provide a more comprehensive understanding of the safety of CAR T-cell therapies in a real-world setting, which can guide clinical decision-making, inform patient management strategies, and contribute to post-market surveillance efforts. Each of these is essential to promoting and ensuring long-term patient safety after treatment with CAR T-cell therapy.

## Patients and Methods

### Study Design

This retrospective, observational study utilized data from the FAERS database to characterize the safety profile of two FDA-approved CAR T-cell therapies, liso-cel and axi-cel, for DLBCL. Specifically, case reports of AEs of interest (ie, CRS, ICANS, cytopenia, and infections) and rare and life-threatening complications (ie, seizure and hemophagocytic lymphohistiocytosis) were compared. This study also examined the use of different concomitant treatments to better understand the impact of liso-cel and axi-cel on the need for supportive therapy.

### Data Source

The FAERS database is publicly available and consists of anonymized drug safety reports spontaneously submitted by health care professionals, patients, and pharmaceutical manufacturers. Although submission is voluntary from patients and health care professionals, it is a regulatory requirement for manufacturers to submit reports to the FDA if they receive a submission directly from patients or health care professionals. Case reports typically include patient demographic characteristics, suspected involved drugs (primary and/or secondary involvement), and patient outcomes. All AEs in the FAERS database are coded according to the Medical Dictionary for Regulatory Activities terminology (ie, Preferred Terms) based on distinct descriptors such as signs and symptoms. At the analysis cut-off date, FAERS data files were released quarterly by the FDA.

### Inclusion and Exclusion Criteria

Case reports spanning Q4 2017 through Q3 2024 were extracted and assessed to cover the period from product approval to the data cut-off for the current analysis. Only cases that identified liso-cel or axi-cel as the primary suspect therapy for adverse reactions and as the indicated treatment used for DLBCL were included. Duplicate reports from different sources of reporting (eg, patient and drug manufacturer) were excluded from the analysis.

### Data Processing and Deduplication

Data identified for inclusion in the analysis underwent deduplication steps as recommended by the FDA.^13^ The first deduplication step identified the most up-to-date case report by identifying case reports with the same case ID and keeping the most recent case report based on its follow-up number, event date, case version, submission year, and quarter. The second deduplication step was conducted to identify duplicate case reports that came from different reporters (eg, patient, clinician, or manufacturer). Case reports involving the same drugs and reactions were considered duplicates if they matched on all or all but one of event date, gender, age, country, drug start dates, and indications. Only the case report with the most recent follow-up was kept among duplicates.

After deduplication and filtering to the drugs of interest (ie, liso-cel and axi-cel), the corresponding drug indications were extracted and reviewed to determine if they were relevant to DLBCL. Any AEs with a minimum of 50 occurrences in the deduplicated dataset were included and mapped to the AEs of interest. This inclusion threshold was employed as a pragmatic approach to ensure statistical relevance of included AEs and to reduce noise from rare or potentially spurious reports. Case reports that were missing the month or day in drug initiation dates were imputed by assigning the first month of the year when the month was missing and the first day of the month when the day was missing.

### Descriptive Analysis

Demographic characteristic variables from FAERS were summarized by case reports stratified by those that listed liso-cel or axi-cel as a primary suspect therapy for AE. Differences in distributions of characteristics between liso-cel and axi-cel case reports were measured using absolute standard mean differences. A standardized mean difference (SMD) ≥0.1 was considered indicative of potentially important imbalances between liso-cel and axi-cel case reports. In addition to comparing demographic characteristics, frequencies of AEs and outcomes of case reports were summarized.

### Primary Analysis

The primary analysis assessed the relative frequency of AEs associated with liso-cel and axi-cel in patients with DLBCL. Disproportionality analyses were performed to compare the frequencies of AEs between the 2 therapies. For each AE, reporting odds ratios (ROR) and 95% CI were calculated. The ROR was used to measure the disproportionality between therapies, with an ROR >1 indicating that liso-cel had greater odds of an AE compared with axi-cel, and an ROR <1 indicating that liso-cel had lower odds of an AE than axi-cel. In addition, adjusted RORs were estimated using a logistic regression model adjusting for age and sex to compare reporting frequencies after accounting for differences in the patient population. All statistical analyses were conducted using R software (version 4.4.1).^14^

### Sensitivity Analyses

To account for differences in FDA approval dates and thus follow-up periods, the following 2 sensitivity analyses were performed: (1) analysis of case reports after FDA approval of liso-cel, wherein the inclusion period was defined as February 5, 2021, to September 2024, and (2) analysis of case reports within 2 years of FDA approval for each drug. Only case reports that contained drug initiation dates were included in the sensitivity analyses.

Furthermore, a sensitivity analysis adjusting for differences in patient age and sex was performed to determine the influence of these characteristics on AE reporting frequency. Adjustment was done using a multivariable logistic regression model with drug, age, and sex as the independent variables and the AE of interest as the dependent variable. Age was recorded as a binary variable to indicate whether a case report was in a patient older or younger than 65 years.

### Concomitant Treatment Use

Use of concomitant treatments was also investigated among liso-cel and axi-cel case reports submitted to FAERS. Concomitant treatments were identified as any medication other than liso-cel or axi-cel explicitly listed as concomitant in the FAERS case report. For specific concomitant treatments of interest, all relevant drug identifiers (eg, generic, brand, or investigational drug names) were compiled to match these terms within the FAERS database. The proportion of submissions reporting one or more concomitant therapies of any kind was calculated across all included case reports and for liso-cel and axi-cel, respectively. Use of common concomitant treatments (ie, corticosteroids, intravenous immunoglobulin (IVIG), and tocilizumab) and concomitant treatment specific for infections and ICANS (ie, azole antifungal agents and levetiracetam, respectively) by patients receiving liso-cel or axi-cel were examined.

### Ethics Statement

The FAERS database is publicly accessible and anonymizes and de-identifies patient records. Consequently, ethical clearance and informed consent are exempted for this study.

## Results

### Characteristics of AE Reports Related to CAR T-Cell Therapy in DLBCL

Approximately 17,000,000 case reports were identified in FAERS between Q4 2017 and Q3 2024. After deduplicating and filtering case reports to those with an indication of DLBCL where liso-cel or axi-cel was the primary suspect therapy of AE, there were a total of 12,388 AEs in 3251 unique case reports: 232 (7.1%) for liso-cel and 3019 (92.9%) for axi-cel (Figure 1).

**Figure 1:**
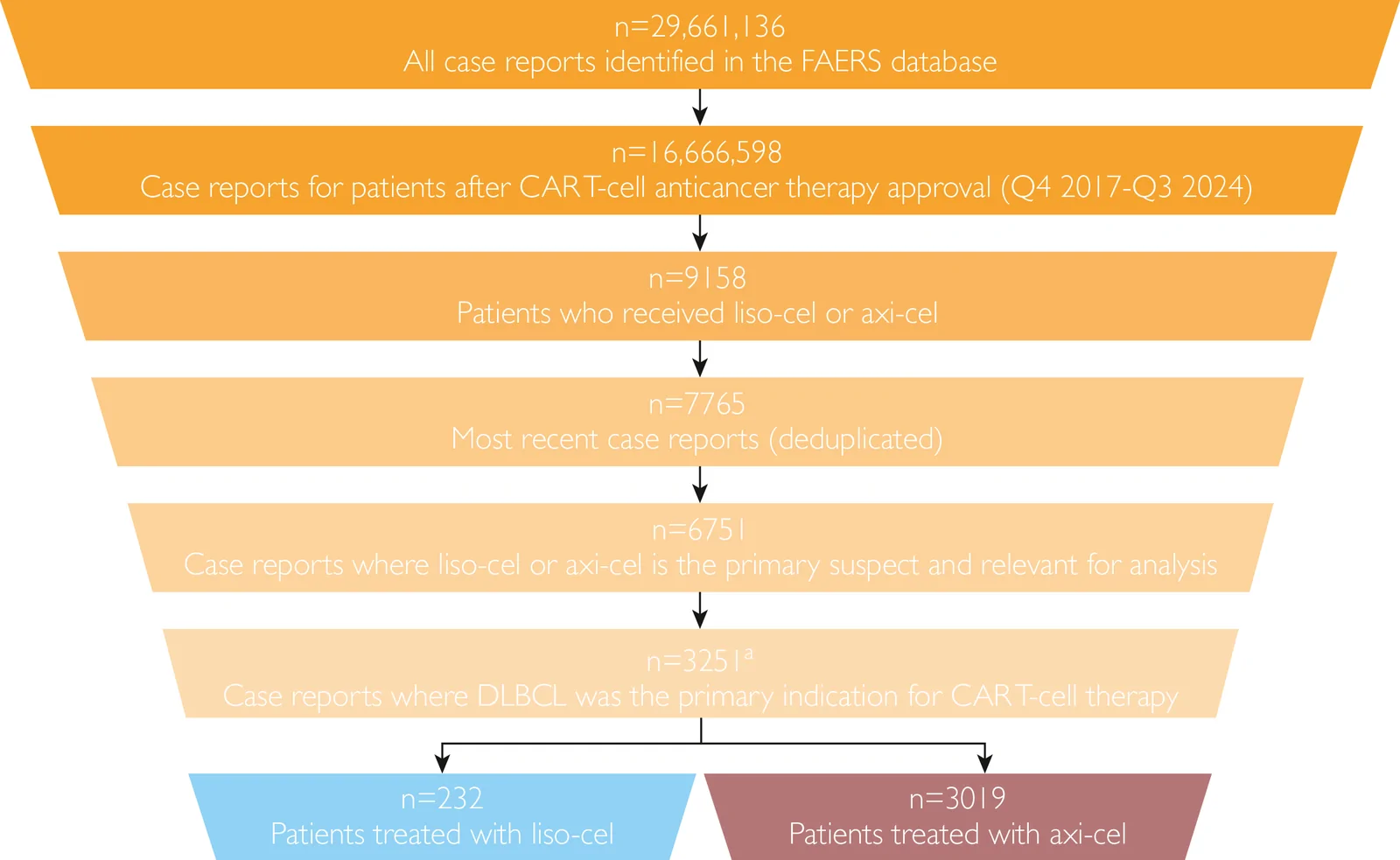
Flow diagram to identify relevant case reports in FAERS database. AE, adverse event; axi-cel, axicabtagene ciloleucel; CAR, chimeric antigen receptor; DLBCL, diffuse large B-cell lymphoma; FAERS, Food and Drug Administration Adverse Event Reporting System; liso-cel, lisocabtagene maraleucel; Q, quarter. ^a^Total AEs, n=12,388.

Overall, only minor demographic differences were observed among the included case reports that identified liso-cel or axi-cel as the primary suspect therapy of AE (Table 1). The proportions of female patients and average weights were similar between the liso-cel and axi-cel reports. However, differences were noted in the distributions of age, geographic region, and the type of case report (30-day, 5-day, direct, expedited—15-day, periodic—nonexpedited). The mean age of patients described in the case reports for liso-cel was about 9 years older than those reported for axi-cel (67.9 years vs 58.9 years; SMD=0.73). There were fewer case reports outside of North America and Europe for liso-cel than for axi-cel (0% vs 4.4%; SMD=0.44). A smaller proportion of liso-cel than axi-cel case reports were classified as expedited report types (32.3% vs 53.6%; SMD=0.57).

**Table 1:** Case Report Demographic Characteristics^a^

Characteristic	Liso-cel n=232	Axi-cel n=3019	Absolute standardized mean difference^b^
Y, n (%)			1.10
2017	0	1 (<0.1)	
2018	1 (0.4)	312 (10.3)	
2019	0	432 (14.3)	
2020	0	436 (14.4)	
2021	38 (16.4)	324 (10.7)	
2022	65 (28.0)	468 (15.5)	
2023	63 (27.2)	557 (18.5)	
2024	65 (28.0)	489 (16.2)	
Quarter, n (%)			0.12
Quarter 1 (January-March)	57 (24.6)	678 (22.5)	
Quarter 2 (April-June)	52 (22.4)	724 (24.0)	
Quarter 3 (July-September)	55 (23.7)	846 (28.0)	
Quarter 4 (October-December)	68 (29.3)	771 (25.5)	
Sex, n (%)			0.04
Female	83 (35.8)	1090 (36.1)	
Male	140 (60.3)	1681 (55.7)	
Unknown	9 (3.9)	248 (8.2)	
Age group, n (%)			0.82
<65 y	55 (23.7)	1498 (49.6)	
65-75 y	86 (37.1)	701 (23.2)	
≥75 y	55 (23.7)	17 (0.6)	
Unknown	36 (15.5)	647 (21.4)	
Age (y), mean ± SD	67.9 ± 11.1	58.9 ± 13.7	0.73
Unknown, n (%)	22 (9.5)	566 (18.4)	
Weight, kg, mean ± SD	78.5 ± 19.8	79.7 ± 21.7	0.06
Unknown, n (%)	82 (35.3)	1468 (48.6)	
Reporter region, n (%)			0.44
Africa	0	1 (<0.1)	
Asia	0	73 (2.4)	
Australasia	0	55 (1.8)	
Europe	34 (14.7)	789 (26.1)	
North America	198 (85.3)	2098 (69.5)	
South America	0	3 (0.1)	
Initial or follow-up, n (%)			0.06
Follow-up case report	59 (25.4)	854 (28.0)	
Initial case report	173 (74.6)	2165 (71.7)	
Type of report submitted, n (%)			0.57
30 d^c^	0	0	
5 d^c^	0	0	
Direct^d^	117 (50.4)	735 (24.4)	
Expedited (15 d)^e^	75 (32.3)	1619 (53.6)	
Periodic (nonexpedited)^e^	40 (17.2)	665 (22.0)	

Proportions and means were calculated among case reports with no missing data.

a Abbreviations: Axi-cel, axicabtagene ciloleucel; FDA, United States Food and Drug Administration; liso-cel, lisocabtagene maraleucel.

b Standardized mean differences were estimated using methods described in Austin 2009.^30^

c Reports are voluntarily submitted to the FDA by nonmanufacturers.

d Reports are combination product reports (combination products are considered both drug and device)

e Reports are from manufacturers.

### Primary Analysis

In the disproportionality analysis, liso-cel had a significantly lower ROR (95% CI) for CRS (0.68; 0.52-0.89), ICANS (0.62; 0.47-0.82), and cytopenia (0.41; 0.24-0.71) than axi-cel (Figure 2). Similar results were observed for infections but were not statistically significant (ROR 0.73; 95% CI 0.40-1.33; *P*=.31).

**Figure 2:**
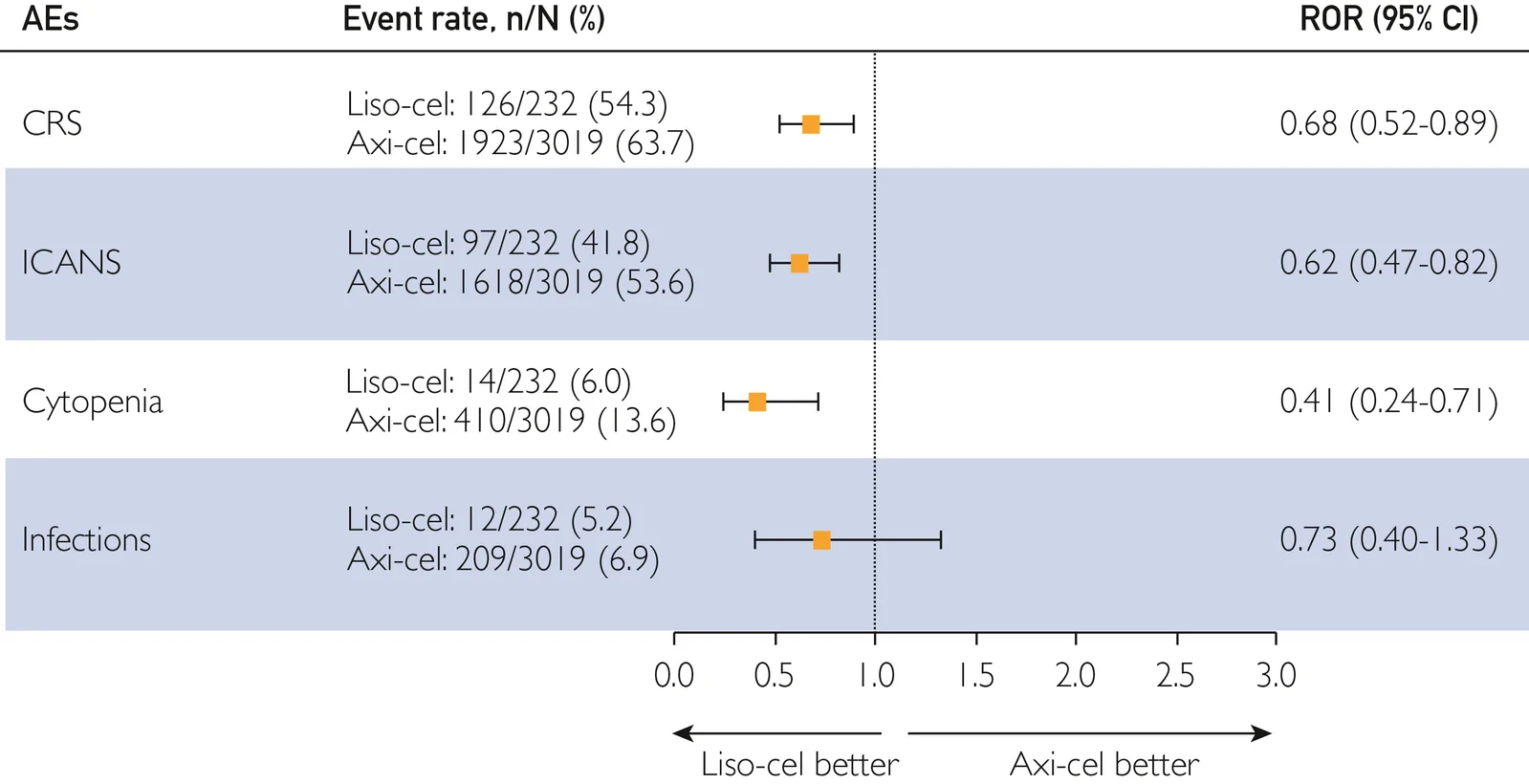
Primary disproportionality analysis of AEs between liso-cel and axi-cel. Results are calculated using data from the FAERS database between Q4 2017 and Q3 2024. ROR is used to determine if a drug has a disproportionate number of events compared with other drugs. An ROR >1 indicates that liso-cel had greater odds of an AE than axi-cel. An ROR <1 indicates that liso-cel had lower odds of an AE than axi-cel. AE, adverse event; axi-cel, axicabtagene ciloleucel; CRS, cytokine release syndrome; FAERS, Food and Drug Administration Adverse Event Reporting System; ICANS, immune effector cell–associated neurotoxicity syndrome; liso-cel, lisocabtagene maraleucel; Q, quarter; ROR, reporting odds ratio.

### Sensitivity Analyses

Sensitivity analyses adjusting for differences in the post-market approval time frame (ie, AEs limited to after liso-cel approval or within 2 years of treatment approval) yielded results consistent with the primary analysis (Table 2). Findings across both sensitivity analyses were similar, indicating that liso-cel was associated with significantly lower reporting odds for CRS(2-year post-drug approval sensitivity analysis, *P*<.001; post liso-cel approval sensitivity analyis, *P*=.003), ICANS(*P*=.003; *P*=.003), and cytopenia (*P*=.01; *P*=.02) compared with axi-cel. No differences were observed in the reporting of infections between liso-cel and axi-cel case reports (Table 2). The sensitivity analysis adjusting for patient age and sex also found results that were similar to the primary analysis (Table 3).

**Table 2:** Sensitivity Disproportionality Analysis of AE Reporting Between Liso-cel and Axi-cel Adjusting for Differences in the Post-Market Approval Time Frame^a^

AE	Treatment arm	Post–Liso-Cel approval sensitivity analysis^b^		Two-year post-drug approval sensitivity analysis^c^	
		Event rate, n/N (%)	ROR (95% CI)	Event rate, n/N (%)	ROR (95% CI)
CRS	Liso-cel	114/198 (57.6)	**0.63 (0.46-0.85)**	68/122 (55.7)	**0.49 (0.34-0.73)**
	Axi-cel	821/1200 (68.4)		586/816 (71.8)	
ICANS	Liso-cel	88/198 (44.4)	**0.63 (0.46-0.85)**	53/122 (43.4)	**0.49 (0.33-0.72)**
	Axi-cel	672/1200 (56.0)		498/816 (61.0)	
Cytopenia	Liso-cel	13/198 (6.6)	**0.50 (0.28-0.90)**	8/122 (6.6)	**0.38 (0.18-0.81)**
	Axi-cel	148/1200 (12.3)		126/816 (15.4)	
Infections	Liso-cel	12/198 (6.1)	0.89 (0.48-1.67)	7/122 (5.7)	0.75 (0.34-1.69)
	Axi-cel	81/1200 (6.8)		61/816 (7.5)	

a Abbreviations: AE, adverse event; axi-cel, axicabtagene ciloleucel; CI, confidence interval; CRS, cytokine release syndrome; ICANS, immune effector cell–associated neurotoxicity syndrome; liso-cel, lisocabtagene maraleucel; ROR, reporting odds ratio.

b Includes case reports where the individual started taking the drug of interest after liso-cel approval.

c Includes case reports where the individual started taking their drug of interest within 2 years of the drug’s FDA approval date. ROR is used to determine if a drug has a disproportionate number of events compared with other drugs. An ROR >1 indicates that liso-cel had greater odds of an AE than axi-cel. An ROR <1 indicates that liso‑cel had lower odds of an AE than axi-cel. Bolding/Bolded print indicates statistically significant values.

**Table 3:** Sensitivity Disproportionality Analysis of AE Reporting for Liso-cel Compared With Axi-Cel Adjusted for Patient, Age, and Sex

AE	Adjusted results, ROR (95% CI)
CRS	**0.61 (0.46-0.82)**
ICANS	**0.43 (0.32-0.57)**
Cytopenia	**0.44 (0.24‒0.75)**
Infections	0.67 (0.33-1.20)

Results are calculated using data from the FAERS database between Q4 2017 and Q3 2024. Case reports missing data for age and/or sex were excluded from this sensitivity analysis. ROR is used to determine if a drug has a disproportionate number of events compared with other drugs. An ROR >1 indicates that liso-cel had greater odds of an AE than axi-cel. An ROR <1 indicates that liso-cel had lower odds of an AE than axi-cel. Bolding/Bolded print indicates statistically significant values.

Abbreviations: AE, adverse event; axi-cel, axicabtagene ciloleucel; CI, confidence interval; CRS, cytokine release syndrome; FAERS, Food and Drug Administration Adverse Event Reporting System; ICANS, immune effector cell–associated neurotoxicity syndrome; liso-cel, lisocabtagene maraleucel; Q, quarter; ROR, reporting odds ratio.

### Concomitant Treatment Use

The proportion of cases reporting use of at least one concomitant medication was ∼38% lower for liso-cel than axi-cel (21.1% vs 33.8%; *P*<.001) (Table 4). Concomitant use of corticosteroids was 37% lower for liso-cel than axi-cel (4.7% vs 7.5%); concomitant use of azoles to treat fungal infections was 49% lower for liso-cel than axi-cel (4.3% vs 8.5%); concomitant use of levetiracetam was 58% lower for liso-cel than axi-cel (4.3% vs 10.2%); concomitant use of tocilizumab was similar for liso-cel and axi-cel (3.5% vs 3.2%). Use of IVIG was comparatively rare (<1%) for both CAR T-cell therapies.

**Table 4:** Summary of Concomitant Drug Use Between Liso-Cel and Axi-Cel

Concomitant Therapy	All Case Reports N=3251	Liso-cel n=232	Axi-cel n=3019
Any concomitant therapy, n (%)	1068 (32.9)	49 (21.1)	1019 (33.8)
Concomitant corticosteroids, n (%)	236 (7.3)	11 (4.7)	225 (7.5)
Concomitant azole (antifungal agents), n (%)	266 (8.2)	10 (4.3)	256 (8.5)
Concomitant levetiracetam (antiepileptic agent), n (%)	319 (9.8)	10 (4.3)	309 (10.2)
Concomitant tocilizumab, n (%)	105 (3.2)	8 (3.5)	97 (3.2)
Concomitant IVIG, n (%)	13 (0.4)	2 (0.9)	11 (0.4)

Abbreviations: Axi-cel, axicabtagene ciloleucel; IVIG, intravenous immunoglobulin; liso-cel, lisocabtagene maraleucel.

## Discussion

This study used the FAERS database to compare safety profiles of the 2 most common CD19-directed CAR T-cell therapies, liso-cel and axi-cel, for the treatment of DLBCL. Our analysis of real-world AE data revealed distinct safety differences between the 2 therapies, particularly in the frequency of CRS, ICANS, and cytopenia. Our analysis was limited to safety and did not include efficacy.

Overall, fewer case reports identified liso-cel as the primary suspect therapy of AEs compared with axi-cel. As axi-cel had a longer period to accumulate AE reports (liso-cel was approved after axi-cel), sensitivity analyses were conducted to account for differences in reporting time frames. These sensitivity analysis results were consistent with the primary analysis, indicating that the reporting time frame was not the sole driver of the observed difference and that there is an overall lower frequency of AEs reported among patients receiving liso-cel than axi-cel.

The primary and sensitivity analyses in this study indicated that liso-cel was associated with significantly fewer reports for CRS, ICANS, and cytopenia than axi-cel. These results align with the known clinical profiles of these drugs, where liso-cel has been associated with a lower incidence of severe CRS and ICANS compared with axi-cel.^15^^,^^16^ Despite differences in AE definitions between this study and other observational studies that analyzed FAERS data to compare CAR T-cell therapies in DLBCL, axi-cel was shown to have more frequent reporting of ICANS than comparator CAR T-cell therapies.^17^^,^^18^ The findings of this study are also aligned with multiple indirect treatment comparisons, which reported significantly lower odds of all-grade CRS, grade ≥3 CRS, any-grade ICANS, and grade ≥3 ICANS for liso-cel than for axi-cel (all available *P* values are smaller than 0.05).^19^, ^20^, ^21^ In contrast, the incidence of infections was reported at similar frequencies for both therapies, potentially because of lower event rates for infection compared with other AEs included in the analysis. Moreover, infection rates between liso-cel and axi-cel were similar, despite the mean age of the liso-cel cohort being roughly 9 years older on average (SMD=0.46). When adjusting for patient age and sex, the observed difference in infection frequency (favoring liso-cel) became more prominent but did not reach significance (*P*=.21). These findings underscore the importance of continued monitoring for these events.

Concomitant treatment use was less frequently reported in case reports for liso-cel than in those for axi-cel. Overall concomitant treatment use was 38% lower for liso-cel than axi-cel (21.1% vs 33.8%; *P*<.001), ranging from a 37% lower use of corticosteroids to a 58% lower use of levetiracetam than axi-cel. Use of IVIG was reported in <1% of cases for both liso-cel and axi-cel. Lower AE frequencies observed for liso-cel in the FAERS database may help explain the reduced use of concomitant treatment compared with axi-cel. Corticosteroids and tocilizumab are common medications in the management of CRS and ICANS in patients receiving CAR T-cell therapy.^22^, ^23^, ^24^, ^25^ Although corticosteroids were less frequently reported with liso-cel, the use of tocilizumab was similar between liso-cel and axi-cel despite the higher rates of CRS with axi-cel. Similarly, reduced use of azole antifungals for infections and cytopenia in patients undergoing CAR T-cell therapy^23^^,^^26^ may correspond to fewer reports of these events with liso-cel. Although the reporting frequency of infections was not significantly different to that of axi-cel, the higher use of azole antifungals may suggest a greater need for antifungal prophylaxis or management in patients treated with axi-cel.^27^ Levetiracetam, commonly used for the treatment of seizures associated with ICANS after CAR T-cell therapy,^28^ was also less frequently reported with liso-cel compared with axi-cel, aligning with its lower frequency of ICANS.

A key strength of this study is its use of FAERS real-world data, offering broader safety insights beyond the limitations of clinical trials. Other studies have compared the real-world clinical outcomes of liso-cel and axi-cel.^15^^,^^29^ An analysis of the DESCAR-T registry reported similar effectiveness profiles between liso-cel and axi-cel; however, the study was limited to a specific population (ie, second-line cohorts within a French registry) and did not provide an in-depth characterization of safety across broader practice settings.^29^ Similarly, a single-institution analysis reported clinical outcomes in third-line or later settings and in a smaller cohort than our study (n=87), with limited power to fully assess less frequent AEs.^15^ In contrast, our study uses a large, nationally representative real-world dataset of 3251 patients across the United States, enhancing generalizability beyond smaller or single-center cohorts. Moreover, this analysis applied formal disproportionality methods to identify differential safety signals, offering a methodologic and interpretive approach that differs from prior descriptive or outcomes-based studies. Although earlier work focuses primarily on efficacy and survival, our study is specifically designed to characterize AE reporting patterns and strengthen the postmarketing safety evidence base. The alignment of our findings with prior reports supports consistency across independent data sources, thereby improving confidence in these observations.

However, it is important to note that FAERS data has inherent limitations, including potential underreporting or misclassification of AEs, differences in reporting practices, and not capturing the duration of AEs. Moreover, while the database includes indicators of serious outcomes (eg, hospitalization and deaths), these are reported at the case level and cannot be reliably attributed to a specific AE when multiple events are present within a single report. Because of the high degree of missingness in the FAERS database, some analyses (eg, use of concomitant medications) should be interpreted cautiously. One of the limitations of this study is the retrospective nature of the analysis, which prevents the ability to draw definitive conclusions about causality between the CAR T-cell therapies and the observed AEs. In addition, the database does not capture detailed patient and disease characteristics such as AE timing or grade, management strategies, or detailed treatment regimens. Access to these data could further inform our understanding of the safety differences observed between liso-cel and axi-cel. Despite these limitations, the large volume of case reports available in FAERS strengthens the reliability of the findings. Future studies, including prospective observational studies or registry data, are needed to validate these findings and explore potential mechanisms underlying the observed safety differences. Furthermore, this study did not include analyses of manufacturing timelines or delays, or of efficacy, which are also important parameters in clinical decision-making.

## Conclusion

This study provides valuable insights into the safety profiles of the 2 most common CAR T-cell therapies for DLBCL in a real-world setting. The findings suggest that liso-cel may be associated with a more favorable safety profile than axi-cel, particularly in terms of CRS, ICANS, and cytopenia. These results contribute to the growing body of evidence on the comparative safety of CAR T-cell therapies, showing differences in the tolerability profile of liso-cel, which can aid clinicians in making informed treatment decisions for patients with DLBCL.

## Potential Competing Interests

Dr Elsallab reports honoraria and speakers’ bureau for Miltenyi Biotec; consulting or advisory roles for Exthymic; and is listed as an inventor on patents related to adoptive cell therapies held by Massachusetts General Hospital. Dr Lunning reports consulting or advisory roles for AbbVie, ADC Therapeutics, Astellas Pharma, AstraZeneca, Bristol Myers Squibb, Daiichi Sankyo/Lilly, EUSA Pharma, Fate Therapeutics, Genentech, Genmab, Ipsen, Kite Pharma (a Gilead Company), Loxo Oncology, Novartis, Nurix Therapeutics, Pharmacyclics, Regeneron, Seagen, and Takeda; and research funding from Celgene, a Bristol Myers Squibb Company, and Curis. Dr Ellithi has no conflicts of interest to declare. Farah Toron, Drs Braun and Lin Wang are employees of Bristol Myers Squibb and hold shares. Pearl Wang and Maxwell Jones are employees of EVERSANA, which received funding from Bristol Myers Squibb for this publication. Dr Maus received research funding from Kite Pharma (a Gilead Company), Moderna, and Sobi; membership on the Board of Directors for 2seventy bio; and is listed as an inventor on patents related to adoptive cell therapies held by Massachusetts General Hospital (some licensed to ProMab and Luminary) and the University of Pennsylvania (some licensed to Novartis).

## Ethics Statement

The FAERS database is publicly accessible and anonymizes and de-identifies patient records. Consequently, ethical clearance and informed consent are exempted for this study.
